# Protective potential effects of hydroalcoholic extract of *Teucrium polium* L. (Lamiaceae) against paraquat-induced lung fibrosis: An experimental study in rats

**DOI:** 10.22038/AJP.2023.22121

**Published:** 2023

**Authors:** Shahrzad Molavinia, Mehdi Goudarzi, Mohammad Ebrahim Azemi, Zahra Basir, Zahra Nazari Khorasgani, Mohammad Reza Rashidi Nooshabadi, Rezvan Ebrahimi, Mohammad Javad Khodayar

**Affiliations:** 1 *Medicinal Plant Research Center, Ahvaz Jundishapur University of Medical Sciences, Ahvaz, Iran*; 2 *Toxicology Research Center, Medical Basic Sciences Research Institute, Ahvaz Jundishapur University of Medical Sciences, Ahvaz, Iran*; 3 *Department of Basic Sciences, Faculty of Veterinary Medicine, Shahid Chamran University of Ahvaz, Ahvaz, Iran*; 4 *Department of Pharmacology, Faculty of Pharmacy, Ahvaz Jundishapur University of Medical Sciences, Ahvaz, Iran*; 5 *Department of Toxicology, Faculty of Pharmacy, Ahvaz Jundishapur University of Medical Sciences, Ahvaz, Iran*

**Keywords:** Lung injury, Oxidative stress, Pulmonary fibrosis, Paraquat, Teucrium polium

## Abstract

**Objective:**

Paraquat (PQ) is a highly toxic herbicide that causes pulmonary fibrosis (PF), and no specific antidote is available against it. *Teucrium polium* L. is a plant that exhibits antioxidant and anti-inflammatory activities. The present study evaluates the preventive and therapeutic effects of *T. polium* extract (TPE) against PQ-induced lung fibrosis in rats.

**Materials and Methods:**

We divided rats into five groups of eight. Groups one and two received saline and PQ (20 mg/kg, i.p.), respectively. Groups three to five were treated with TPE (50, 100, and 200 mg/kg, by gavage) started one week before PQ administration and lasted three weeks after PQ administration.

**Results:**

Our findings showed that PQ significantly increased lung malondialdehyde, nitric oxide, hydroxyproline, lung index, Ashcroft score, red blood cells accumulation, and inflammatory cell infiltration. Moreover, PQ decreased catalase and glutathione peroxidase activities and glutathione content. The results of hematoxylin-eosin and Masson's trichrome staining indicated that PQ destroyed lung parenchyma and developed PF (p<0.05 to p<0.001). Gavage with TPE significantly improved biochemical and histological abnormalities induced by PQ in rats (p<0.05 to p<0.001).

**Conclusion:**

The current survey indicated that treatment with TPE could reduce and reverse PQ-induced PF, which may be due to the phenolic compounds present in TPE.

## Introduction

Paraquat (PQ: 1,10-dimethyl-4,40-bipyridinium dichloride) is a toxic herbicide extensively used to handle weed control in 130 countries worldwide, specifically in Asia (Xu and Wang, 2016 ▶; Amin et al., 2020 ▶). Ingestion of PQ is the most common cause of severe and sometimes fatal poisoning, whether accidental or intentional (Kanno et al., 2019 ▶). PQ damages the central nervous system, liver, kidney, heart, and gastrointestinal tract, however, pulmonary damage is the leading cause of PQ-induced death (Pourgholamhossein et al., 2018 ▶). This injury is characterized by an initial destructive phase within a few days, leading to damaged cellular components, impaired lung epithelial cell function, pulmonary hemorrhage, interstitial edema, and infiltration of inflammatory cells into alveoli (Tomita et al., 2007 ▶). The second phase lasts a few weeks and, in this phase, PQ causes overproduction of extracellular matrix (ECM) components (such as collagen) within the lung parenchyma, resulting in fibrosis (Li et al., 2015 ▶). Pulmonary fibrosis (PF) is a progressive lung disorder that impairs lung compliance and gas exchange. Hence, eventually, irreversible loss of respiratory function and death occur (Richeldi et al., 2017 ▶). There is no known antidote or treatment against PQ intoxication, though various treatment methods lead to diminished lipid peroxidation and collagen production (Mohammadi-Karakani et al., 2006 ▶).

Previous research has indicated that antioxidants and anti-inflammatory agents are beneficial in preventing and treating fibrosis (Li et al., 2017 ▶). *Teucrium polium* L. (TP) is a Lamiaceae plant found across Europe, North Africa, and Asia. This plant is used widely in the daily diet and traditional medicine (Moghadam and Kharazian, 2020 ▶). *T. polium* extract (TPE) contains compounds with antioxidant properties, such as flavonoids, terpenoids, phenylpropanoids, iridoid glycosides, and several other substances that have a protective role in damage caused by oxidative stress (OS) and dysfunction of cells and tissues (Bahramikia and Yazdanparast, 2012 ▶). Evidence suggests that TP has multiple bioactivities, including anti-oxidation and anti-inflammation properties (Baali et al., 2016 ▶; Rahmouni et al., 2017 ▶), anti-atherosclerosis mechanisms (Nor et al., 2019 ▶), anti-hypertensive effects (Niazmand et al., 2011 ▶), anti-infectious potential (Bahramikia et al., 2022 ▶), and anti-bronchitis activity (De Marino et al., 2012 ▶). Moreover, studies have revealed that TP can improve pain (Abadian et al., 2016 ▶), ulcers (Fallah Huseini et al., 2019 ▶), diabetes (Tabatabaie and Yazdanparast, 2017 ▶), convulsion (Khoshnood-Mansoorkhani et al., 2010 ▶), and cancers (Hashemi et al., 2020 ▶).

To our knowledge, no previous studies have been conducted to evaluate the effect of TPE against PQ-induced PF. We hypothesized that TP might provide therapeutic advantages against PF. Thus, the present study aimed to assess the beneficial health effects of TPE against PQ-induced lung injury in experimental rats.

## Materials and Methods

**Chemicals**

Chloramine T, trichloro acetic acid (TCA), thiobarbituric acid (TBA), 5,5′-dithiobis-(2-nitrobenzoic acid) (DTNB), and PQ were obtained from Sigma Company (USA). Hydrochloric acid (HCl), perchloric acid, and buffered formalin were purchased from Merck Company (Darmstadt, Germany). All the chemicals utilized were of the analytical grades.

**Preparation of the plant extract**

The plant was collected from Larestan in Fars Province, Iran, during the spring of 2014. A voucher herbarium specimen (number voucher: T-0157) was identified and deposited in the Herbarium of the Department of Pharmacognosy, Faculty of Pharmacy, Ahvaz, Iran. The entire plant was shade-dried, broken down into smaller pieces, and steeped in a 70% aqueous ethanol solvent for three days to make the hydroalcoholic extract. After that, the extract was filtered and dried by rotary evaporation (Abdollahi et al., 2003 ▶).

**Animals**

Forty male Wistar rats, weighing 180-250 g, were obtained from the central animal house, Ahvaz Jundishapur University of Medical Sciences (AJUMS), Ahvaz, Iran. The rats were placed under standard conditions (23±2 °C and 12 hr light/dark cycles) with free access to rat feed and water *ad libitum*. The present research was approved by the Animal Ethics Committee of AJUMS (Research number: 9301).

**Experimental design**

The animals were randomly assigned to five experimental and control groups (eight rats per group). Group I received normal saline by gavage daily. Group II received a single dose of PQ (20 mg/kg, i.p.) daily for three weeks. Groups III to V received varying amounts of TPE (50, 100, and 200 mg/kg, by gavage) daily for four weeks. Treatment with TPE began one week before the administration of PQ and lasted three weeks after PQ administration. We selected the doses of PQ and TPE for our treatment protocol based on previous studies (Hasani et al., 2007 ▶; Khodayar et al., 2014 ▶).

**Sample collection**

The rats were anesthetized 21 days after PQ administration with ketamine and xylazine (90/10 mg/kg) (Javadipour et al., 2019 ▶). The lungs (including both lobes) were removed, then rinsed in ice-cold saline solution. The right lung specimens were set in 10% buffered formalin for histological examination, while the left lung snaps were preserved at -70 °C until the analysis of parameters.

**Assessment of lung index and hydroxyproline (HP) content**

The body weight of animals was recorded once a week during the experiment. The lung index was determined using the equation below: 

[lung weight (mg)/body weight (g)] (Huang et al., 2020 ▶).

 Lung HP content was quantified by the colorimetric assay described by Edwards and O'Brien (1980) ▶. Tissue sections were digested for 22 hr at 130 °C with 6 N HCl. Afterward, buffer and 1 ml chloramine T solution were added into tubes. After 20 min, 1.5 ml perchloric acid was poured and test tubes were heated at 60 °C for 20 min to give a red-brown complex. The absorbance was read at 550 nm by a spectrophotometer (UV-1650 PC, Shimadzu, Japan), and the final results are reported as mg/g of lung tissue.

**Assessment of OS markers and nitric oxide (NO) production**

The malondialdehyde (MDA) levels were assessed by Buege and Aust (1978) ▶ method. For this purpose, a 2.5 ml TCA solution was combined with 0.5 ml of homogenized lung tissue. After 10 min, the mixture was centrifuged at 1000 × g. Then, 1 ml TBA 0.67% was added to 1 ml supernatants, and test tubes were immersed in a bath of boiling water to obtain a pink-color solution after heating for 30 min. In the final part, the absorbance of the supernatant was read at 532 nm by a spectrophotometer (UV-1650 PC, Shimadzu, Japan). The final results are reported as nmol/mg protein. 

The amount of glutathione (GSH) in the homogenate was determined using Ellman's reagent (Ellman, 1959 ▶). Briefly, the homogenate samples were precipitated with 1 ml TCA solution and then, centrifuged (1000 × g) for 10 min. After that, supernatant samples reacted with DTNB (0.04%), and the absorbance of the yellow product was recorded at 412 nm using a spectrophotometer (UV-1650 PC, Shimadzu, Japan). The final results are reported as nmol/mg protein. 

The activity of catalase (CAT) in tissue samples was evaluated by the Aebi (1984) ▶ assay. The centrifugation of tissue samples was performed for 20 min at 1200 × g. Then, 50 µl of homogenized tissue was combined with 200 µl sodium phosphate buffer containing 250 µl of H_2_O_2_. Absorption was read using a spectrophotometer (UV-1650 PC, Shimadzu, Japan) at 240 nm. The final results are reported as U/mg protein. 

Glutathione peroxidase (GPx) activity and NO content measurements were conducted in lung tissue using commercial kits (ZellBio GmbH, Germany). The final results of GPx and NO are reported as U/mg protein and nmol/mg protein, respectively.

**Histological studies**

Five-micrometer paraffin-embedded sections were appropriately stained with hematoxylin-eosin (H&E) and Masson's trichrome (MT). Photomicrographs were taken with a light microscope (Olympus BH-2, Japan) and Dino lite lens (Taiwan) using the DinoCapture 2.0 software.

Microscopic slides (five slides per animal) were evaluated to assess the accumulation of red blood cells (RBCs), inflammation, and alveolar thickness. RBC accumulation and inflammatory cell infiltration were scored in four degrees of normal, mild, moderate, and severe (0–3), using the Szapiel score (Szapiel et al., 1979 ▶). The fibrotic changes were scored from 0 to 8 using the Ashcroft score (Ashcroft et al., 1988 ▶). At a magnification of × 100, the mean of five fields was counted for each slide. The fibrotic score was calculated as the average of all fields.

**Statistical analysis**

In this paper, the data are expressed as mean±SD. Variables were compared using one-way ANOVA with Tukey's *post hoc* procedure. While the histological scores were assessed using the non-parametric Kruskal-Wallis’s test. The experimental data were analyzed by SPSS software, and a p-value of 0.05 was considered statistically significant.

## Results

**Effects of TPE on lung index and HP content in the PQ model of lung fibrosis**

Results revealed that PQ alone triggered a considerable increase in the lung weight/body weight ratios in contrast to the saline group (p<0.001), and gavage with TPE (100 and 200 mg/kg) markedly diminished lung index in contrast to the PQ group (p<0.01 and p<0.001, respectively). In addition, collagen accumulation in the lungs was assessed based on the HP content. 

**Figure 1:**
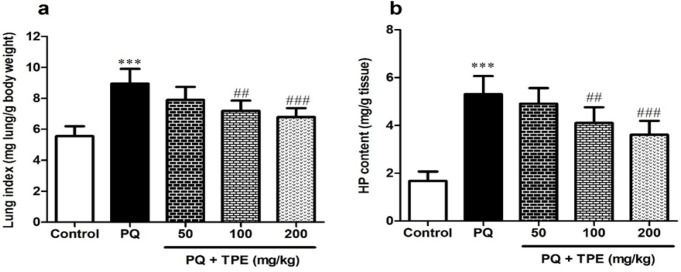
Effect of pretreatment with TPE (50, 100, and 200 mg/kg) on lung index and HP content in rats exposed to PQ (20 mg/kg). Values are means±SD (n=8). PQ: paraquat, TPE: *Teucrium polium *L. extract, HP: hydroxyproline. ***p<0.001 vs. control. ##p<0.01 and ###p<0.001 vs. PQ group

The exposure of PQ significantly increased HP content compared with those of normal rats (p<0.001), and gavage with TPE (100 and 200 mg/kg) substantially attenuated it (p<0.01 and p<0.001, respectively) (Figure 1).

**Effects of TPE on GSH content and CAT and GPx enzyme activities in the PQ model of lung fibrosis**

**Figure 2:**
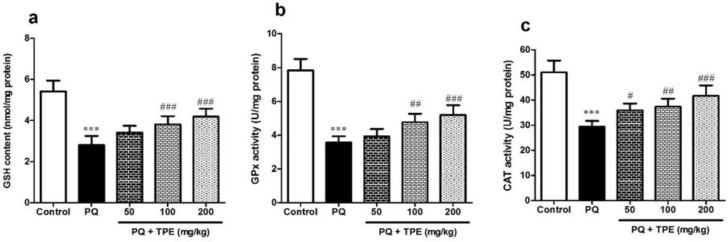
Effect of pretreatment with TPE (50, 100, and 200 mg/kg) on lung tissue levels of GSH and CAT and GPx enzyme activities in rats exposed to PQ (20 mg/kg). Values are means±SD (n=8). PQ: paraquat, TPE: *Teucrium polium *L. extract, GSH: glutathione, GPx: glutathione peroxidase, CAT: catalase. ***p<0.001 vs. control. #p<0.05, ##p<0.01 and ###p<0.001 vs. PQ group

To examine the impact of TPE on OS status, we assessed the GSH content and CAT and GPx enzyme activities. As depicted in Figure 2, the PQ exposure meaningfully lowered the amount of lung GSH and the activity of CAT and GPx versus the control rats (p<0.001). Gavage with TPE (100 and 200 mg/kg) drastically enhanced GSH levels (p<0.001) and activity of CAT and GPx (p<0.01 and p<0.001, respectively) as compared to those administered with PQ alone. However, treated groups with TPE (50 mg/kg) positively affected only CAT activity (p<0.05) while not significantly ameliorating GSH content or GPx activity.

**Effects of TPE on MDA and NO levels in the PQ model of lung fibrosis**

**Figure 3:**
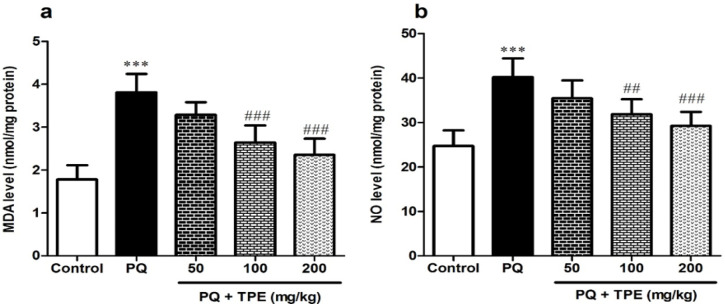
Effect of pretreatment with TPE (50, 100, and 200 mg/kg) on levels of MDA and NO in rats exposed to PQ (20 mg/kg). Values are means±SD (n=8). PQ: paraquat, TPE: *Teucrium polium *L. extract, MDA: malondialdehyde, NO: nitric oxide. ***p<0.001 vs. control. ##p<0.01 and ###p<0.001 vs. PQ group

As shown in Figure 3, PQ caused a marked rise in lung MDA and NO amounts versus the control rats (p<0.001). While gavage with TPE (100 and 200 mg/kg) led to a considerable decrease in MDA (p<0.001) and NO level (p<0.01 and p<0.001, respectively) against the group receiving PQ alone.

**Effects of TPE on histopathological features in the PQ model of lung fibrosis**

**Figure 4.**
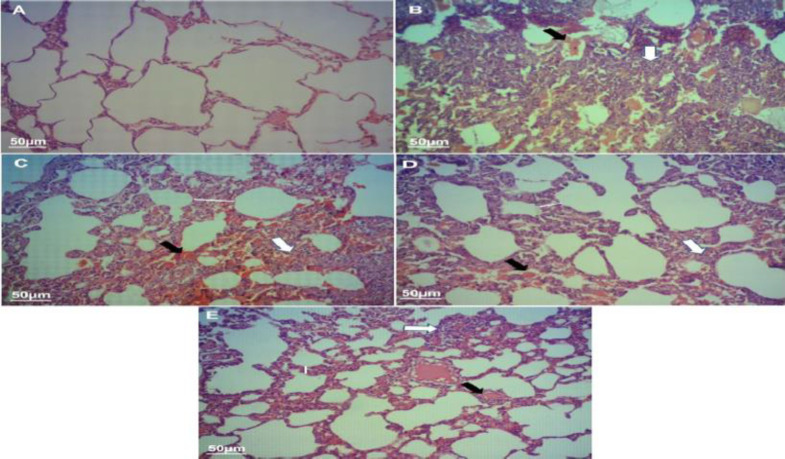
The morphophonological changes of the lung sections were examined by hematoxylin-eosin (H&E) staining (magniﬁcation is 100×). Histopathological appearance of lung tissues in (A) Control, (B) PQ (20 mg/kg/day), (C) PQ (20 mg/kg/day) + TPE (50 mg/kg/day), (D) PQ (20 mg/kg/day) + TPE (100 mg/kg/day), and (E) PQ (20 mg/kg/day) + TPE (200 mg/kg/day) groups. White arrows: infiltration of inflammatory cells; Black arrows: congestion and hemorrhage. PQ: paraquat, TPE: *Teucrium polium *L. extract

**Figure 5:**
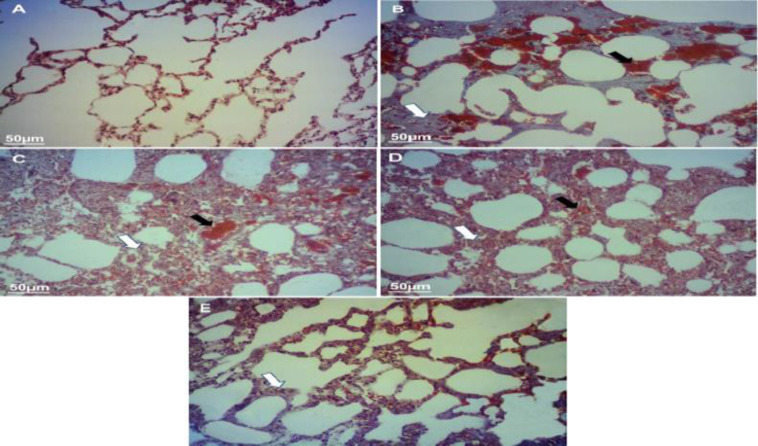
The morphophonological changes of the lung sections were examined by Masson's trichrome (MT) staining (magniﬁcation is 100×). Histopathological appearance of lung tissues in (A) Control, (B) PQ (20 mg/kg/day), (C) PQ (20 mg/kg/day) + TPE (50 mg/kg/day), (D) PQ (20 mg/kg/day) + TPE (100 mg/kg/day), and (E) PQ (20 mg/kg/day) + TPE (200 mg/kg/day) groups. White arrows: accumulation of collagen fibers; Black arrows: congestion and hemorrhage. PQ: paraquat, TPE: *Teucrium polium *L. extract

Histopathologic examination of the lungs in the group treated only with PQ showed lesions, hemorrhage, inflammatory cell infiltration, and alveolar thickness (Figures 4 and 5). The lungs of the control animals were normal, with a thin alveolar wall and no lesions. In the group TPE50 + PQ, no significant pathological changes were observed compared with the PQ group, and features of the lungs were almost similar to the PQ group. Collapsed alveoli, inflammation, RBCs accumulation, and thickness of the alveolar wall were visualized in the treated group by PQ. Both TPE 100 and 200 mg/kg groups improved tissue damage so that the alveoli and the wall between them were similar to the control group. The findings revealed that treatment with TPE notably reduced PQ-induced lung fibrotic lesions.

**Figure 6:**
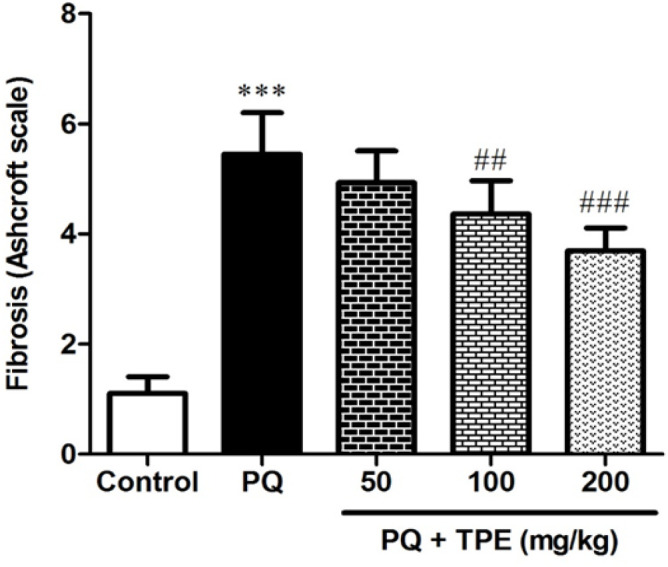
Effect of pretreatment with TPE (50, 100, and 200 mg/kg) on inhibition of fibrotic lesions (Ashcroft score) in rats exposed to PQ (20 mg/kg). Values are means±SD (n=8). PQ: paraquat, TPE: *Teucrium polium *L. extract. ***p<0.001 vs. the control group. ##p<0.01 and ###p<0.001 vs. the PQ only group

As shown in Figure 6, gavage with TPE (100 and 200 mg/kg) reduced the fibrotic lesions in several parts of lung tissue (p<0.01 and p<0.001, respectively). Moreover, amelioration of inflammatory processes in alveolar spaces and accumulation of RBC were observed in the group treated with TPE 100 and 200 mg/kg (p<0.05, Table 1). Gavage with TPE 50 mg/kg showed no significant changes compared with the group receiving PQ only.

**Table 1:** Histopathological parameters in different groups

	**Control**	**PQ**	**PQ + TPE50**	**PQ + TPE100**	**PQ + TPE200**
**Accumulation of RBCs**	1.2±0.34	2.45±0.52^a^	2.13±0.38^a^	1.86±0.31^a, b^	1.59±0.12 ^b^
**Infiltration of inflammatory cells**	1.1±0.11	2.38±0.25 ^a^	2.33±0.18 ^a^	1.75±0.21 ^a, b^	1.68±0.14 ^a,b^

PQ: paraquat at dose of 20 mg/kg. TPE: *Teucrium polium *L. extract. RBCs: red blood cells. ^a^p<0.05 vs. the control group. ^b^p<0.05 vs. the PQ group

## Discussion

This study aimed to determine how TPE affects PQ-induced lung damage in rats. OS and inflammation alterations occur in the early phase of PQ treatment (until day seven). Fibrosis develops in the terminal stages of lung damage. The final phase (fibrotic) lasts up to 14 and 21 days after PQ administration (Chua et al., 2005 ▶). In a similar research project, PQ 20 mg/kg led to lung destruction, and PF occurred after 21 days (Javad-Mousavi et al., 2016 ▶). Hence, we evaluated fibrotic lesions on day 21 after PQ exposure. PQ has a slow clearance rate, leading to the deposit of ECM and malfunction of the lungs. In addition, it leads to OS and cellular component damage through the production of active oxygen species and lipid peroxidation (Suntres, 2002 ▶; Amin et al., 2021 ▶). An overproduction of MDA indicates lipid peroxidation in lung injury after PQ (Mirzaee et al., 2019 ▶). NO is an endogenous free radical that causes inflammation and OS. Zhu et al. (2013) ▶ have reported that NO level increases in PF. Our findings revealed that PQ increased MDA and NO lung tissue levels. In this study, we investigated the endogenous antioxidant system, which contains both enzyme- and non-enzyme-dependent antioxidants. The present research demonstrated that PQ reduces lung tissue antioxidant levels, which corroborates previous research (Zhang et al., 2020 ▶; Ijaz et al., 2021 ▶). The edema formation is associated with collagen accumulation which causes PF (Fouad and Mousa, 2021). The lung index is a crucial marker of edema, and HP content is the main index of collagen accumulation in fibrosis. In line with previous research, our results showed PQ induces edema and collagen deposition (Xu et al., 2014 ▶; Kalantar et al., 2018 ▶).

**Figure 7:**
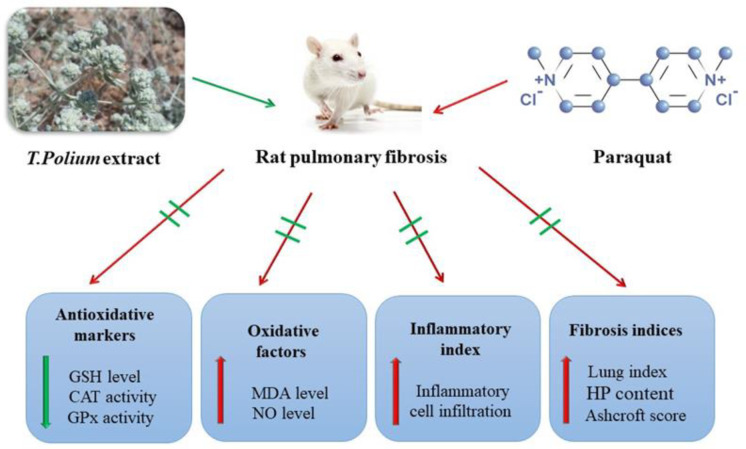
A graphical abstract of the protective effects of *T. polium* extract (50, 100, and 200 mg/kg) on paraquat (20 mg/kg) induced pulmonary injury in rats

In this study, we hypothesized that TPE inhibits injury progression through its antioxidant properties. In an *in vivo* rat model, the antioxidant activity of TPE at three different concentrations of 50, 100, and 200 mg/kg after two weeks was investigated. The results of this study are consistent with our findings which showed that TPE dramatically inhibited OS in rats (Hasani et al., 2007 ▶). As previously reported, treatment with TPE (doses of 250 and 500 mg/kg) resulted in hepatoprotective effects by reducing oxidative damage in a mouse model of hepatitis. Besides, biochemical and histopathological results confirmed that TPE has no toxic effect on the liver in the mentioned doses (Forouzandeh et al., 2013 ▶). In a carbon tetrachloride model of liver toxicity in rats, TPE at a dose of 200 mg/kg by gavage for seven days increased the expression of CAT, SOD, and GPx enzymes and decreased the damage in the animals (Rahmouni et al., 2018 ▶). The present findings are consistent with other research. As schematically shown in Figure 7, TPE positively affected PQ-induced PF in rats. Our data confirmed that TPE has antioxidant activity, and treatment with doses of 100 and 200 mg/kg noticeably ameliorated the MDA, NO, and antioxidant levels. The current study found that TPE exerts antifibrotic effects by attenuating lung index, HP deposition, accumulation of RBCs, inflammatory cell infiltration, and fibrotic lesions. Results indicated that TPE (doses of 100 and 200 mg/kg) presented significant improvement against the destructive effects of PQ on the lungs. However, there were no significant differences between the PQ alone and PQ+TPE 50 mg/kg groups, suggesting that TPE at this dose did not alleviate oxidative damage or fibrotic lesions.

Our research had some limitations, which should be addressed in further studies. One of the study's limitations was the lack of investigation of the inflammation and fibrosis pathways. We did not assess pro-inflammatory and pro-fibrotic cytokines in PF, including interleukins and transforming growth factor-β1. In addition, further studies should measure levels of fibrosis markers such as alpha-smooth muscle actin in the lung tissue. As a result, more research is required to determine the cellular and biological mechanisms underlying TPE's therapeutic effect. However, this research was a preliminary study.

*T. polium* is a perennial herbaceous plant with anti-inflammatory, antioxidant, and several other properties (Amraei et al., 2018 ▶). Nevertheless, several studies have shown that this plant can be hazardous in the early stages of fetal development (Al-Qahdi et al., 2019 ▶) and has adverse effects on male fertility (Al-Tikriti et al., 2017 ▶). Likewise, some studies have shown hepatotoxicity in humans and laboratory animals (Savvidou et al., 2007 ▶; Mimidis et al., 2009 ▶). A further limitation of our study is that we did not assess the safety of TPE. The correlation between increasing doses and adverse effects is unclear. In addition, medicinal plants may contain hundreds to thousands of biologically active compounds and toxic contaminants. However, assessing the safety of these plants is challenging due to their complex nature and species diversity (Albadr et al., 2022 ▶). Hence, more experimentation is needed to identify the exact mechanism of the plant and the type of plant species consumed.

This study showed that the hydroalcoholic extract of TP noticeably ameliorated the lung injury caused by PQ, depending on the dose. However, further experimental investigations are required to estimate the efficacy and safety of TPE for therapeutic and clinical applications. We believe that our research will serve as a base for future studies on interstitial lung diseases.

## Conflicts of interest

The authors have declared that there is no conflict of interest.
